# Supplementary material for: A leafhopper saliva protein mediates horizontal transmission of viral pathogens from insect vectors into rice phloem
Source: Commun Biol. 2022 Mar 4;5:204. doi: 10.1038/s42003-022-03160-y (PMC8897447; doi:10.1038/s42003-022-03160-y)
Supplement: Supplementary file 2 — Supplementary Information [file 42003_2022_3160_MOESM2_ESM.pdf]

Supplementary Information for

A leafhopper saliva protein mediates horizontal transmission of viral pathogens from insect vectors into rice phloem

Table of Contents:

Supplementary Figures (Figs. 1-6)	Pages 2-7
Supplementary Figures (Original images for immunoblots)	Pages 8-11
Supplementary Table	Pages 12

1 ACATTGTTAGCAGCTCTACATGACAACGATGTACTCCGTGCTCGCAGTTTTGTGTGTGGCGGTCGCCGCTCAGGCTGAACTAGACCAAAAGTTTGTAACTGACATCTTGGAAACGAAGGG
1 M T T M Y S V L A V L C V A V A A Q A E L D Q K F V T D I L E T K G

121 AGCTCAATATGAACTCTACAAGACATACACAGAGAAGATCAATTTTGATGCCGACCCCAAAGTGGCCGAGAACACCCACAAGGGTCTTAACATCAAAAGAGACGATGAGACGGTCAACAA
35 A Q Y E L Y K T Y T E K I N F D A D P K V A E N T H K G L N I K R D D E T V N K

241 AGGTGACCTTGAATTGTTCTACTGGCATAAAGATACGAAATTTGCAGTGCCGAGGTTCTTCAGAATGATTGACGACAATAAGGACGACAACATTGAGGTTTCAGAACTGAAAGATGACCT
75 G D L E L F Y W H K D T K F A V P R F F R M I D D N K D D N I E V S E L K D D L

361 AAAAAAGAAACACTTCATCGTTGCTTTGTCAGAGTGTCAGTACCTTCGTTCAAAGGGTCTGAAGCAATCCTGCTACGCTGATCAGATCTTGACCTACGTTGCCTTGACTCTGGGGCCGAA
115 K K K H F I V A L S E C Q Y L R S K G L K Q S C Y A D Q I L T Y V A L T L G P N

481 TCACGTCAAGGCCTTCTACAAGTACCTCGACAAGGATGGCAGTGGTAAGATTGATGAGAATGAAGTTAACAACATCGCAAGCCAGAAAAACAAGGACAAGGAGATCACAGCTGAGGAGTT
155 H V K A F Y K Y L D K D G S G K I D E N E V N N I A S Q K N K D K E I T A E E L

601 GGGAGCCTACTACGGAAACGATGGAGTCACGGAGGATGAACTTACGGACTACGTTACCCAAGAAAGCATCTTGGACGACTTCAGAGGATGCCGAGATTCTGCCACCGAGACAGTTGTTTC
195 G A Y Y G N D G V T E D E L T D Y V T Q E S I L D D F R G C R D S A T E T V V S

721 GTTCAAAAACGATTGCCTGCTGGATGAAGTGGTCGAGTACACCAACGACGACCTCACTGACATGGACATCGCCGTCTTGTTCCGTGCCATTGACAAGGATGAAAATGGCTTTATCACGAT
235 F K N D C L L D E V V E Y T N D D L T D M D I A V L F R A I D K D E N G F I T M

841 GAAGGAACTCGAGTTCATTCTGCCAGAAGCCAAGATCCAGGAGATCAAGGACATTATGGAGATCATTGACATGAGCAGCACTCGATCCAGGGACGTCGACTTCGGCGAGTTACGCACCTA
275 K E L E F I L P E A K I Q E I K D I M E I I D M S S T R S R D V D F G E L R T Y

961 CCTCAGATCTCCCACCGTCTTGATTCAGATGGAAAGGGTATGCACCGTGCCCAAGACCACACAGAAGGAGGAAATTGAATGCATTTTCACCCTGTTCAAGAGTCTCATCAAGGACGAGCA
315 L R S P T V L I Q M E R V C T V P K T T Q K E E I E C I F T L F K S L I K D E H

1081 CGACCCCATCTGGCTCGTGGTTCCCGACCTTGACCAACTCATCACCTTCGAGATATTCGACAAAACAGATGACGGCGTTATTAAGAAAGATGAACTTAAAAGTTTCCCTAAGTTTACCGA
355 D P I W L V V P D L D Q L I T F E I F D K T D D G V I K K D E L K S F P K F T E

1201 AGATGCAACAGTCAACAGATTTTTCAAGGAAGCAGACTCTGACGACAATAACGTGGACGACATCGATCTGGAAGAGTTCAACGAGTACGCTGACATTCCCCACATGGTCTATGGAACTGG
395 D A T V N R F F K E A D S D D N N V D D I D L E E F N E Y A D I P H M V Y G T G

1321 AGACTGTCTCTTGACGTCCAGGAAACAGGGAGATAAACGTGGAGAATGCTTCACCACTGAAGTTGGCGACGTTATTGAAAACTTCAAGTTTGTGGAAAATGAAGCCATCGAGCAATGGTT
435 D C L L T S R K Q G D K R G E C F T T E V G D V I E N F K F V E N E A I E Q W F

1441 CAACACCTACGATAAGGACCAGGATGGATTTATCTCTGAAGCCAAGGAAGACCAACTCAAGGAAAAGTTGGGAGGCGATGTCAAGCTTGTAGACTTGTTCTTGGAAGAACTCGGGCAAGG
475 N T Y D K D Q D G F I S E A K E D Q L K E K L G G D V K L V D L F L E E L G Q G

1561 CGGCAACTTGGTATCGCATTTCGAATTCGACTGGTACGTCAAGCAGCAGGACTTTGCAGACTGCTATGAGGACAAGAAGATTGTCGAGGCTGAGACTAAATCGTTTGACGATACACAATT
515 G N L V S H F E F D W Y V K Q Q D F A D C Y E D K K I V E A E T K S F D D T Q L

1681 GAAGTCTCTCGACGCTGAATTGAAGCTGTACATCGAAGAAGAAATGACTGAGTACGACATCGAAGAGTTTTTCGACATTCTGGATGCTGATGATGACCACGACATCGATATTGATGACTT
555 K S L D A E L K L Y I E E E M T E Y D I E E F F D I L D A D D D H D I D I D D F

1801 CAAGCCCAACCCCGCGTCTCATATGATTAAACTTCTTGAGCTGATCGACAAGCAGGAGGCTGAAGGAGACGAAGACACCCCCGAACAACCCAACGTCAACTACAGAGAGTTCTGGCTGTG
595 K P N P A S H M I K L L E L I D K Q E A E G D E D T P E Q P N V N Y R E F W L W

1921 GCTCAACAACGACGCACAAGACGGCAACCAAAAGATCAAGAACATCTTCGAGAAGGAATGCACGAAACCAGAAAAAACTCATTTCGCCGAGATATACTGCTACGTCAAGACCTTCAAGAA
635 L N N D A Q D G N Q K I K N I F E K E C T K P E K T H F A E I Y C Y V K T F K N

2041 CCCAGAGAAATTCACTGCTTTGACTCCCAAATCACAGTAGTTCACTGTAACAACTTATTTAATAAACTTCTCTCAGAACTAAAAAAAAAAAAAAAAAAAAAAAAAAAAAA
675 P E K F T A L T P K S Q

Supplementary Figure 1. Nucleotide sequence of RdCBP and its deduced amino acid sequence.

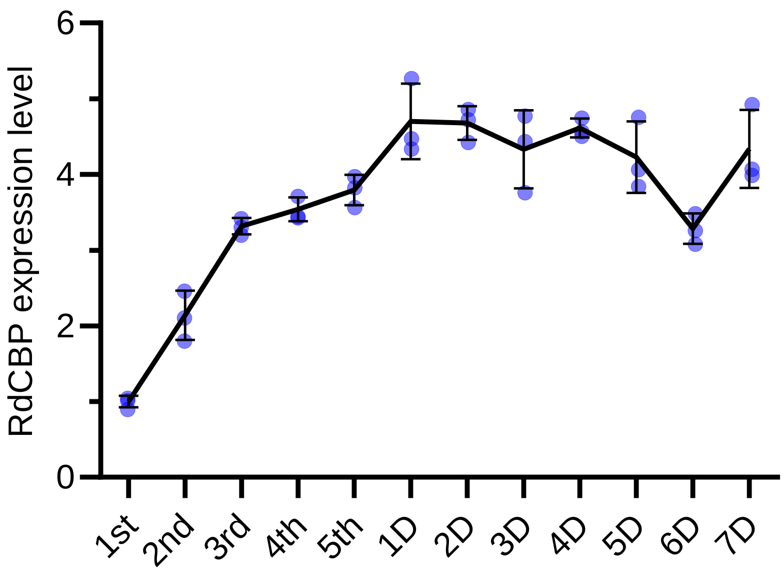

Supplementary Figure 2. The expression levels of RdCBP in salivary glands throughout *R. dorsalis* development

Mean transcript levels of RdCBP at different developmental stages. 1st-5th, first- to fifth-instar larvae; 1-7 d, 1–7 days after eclosion. Bars represent mean ± SD values.

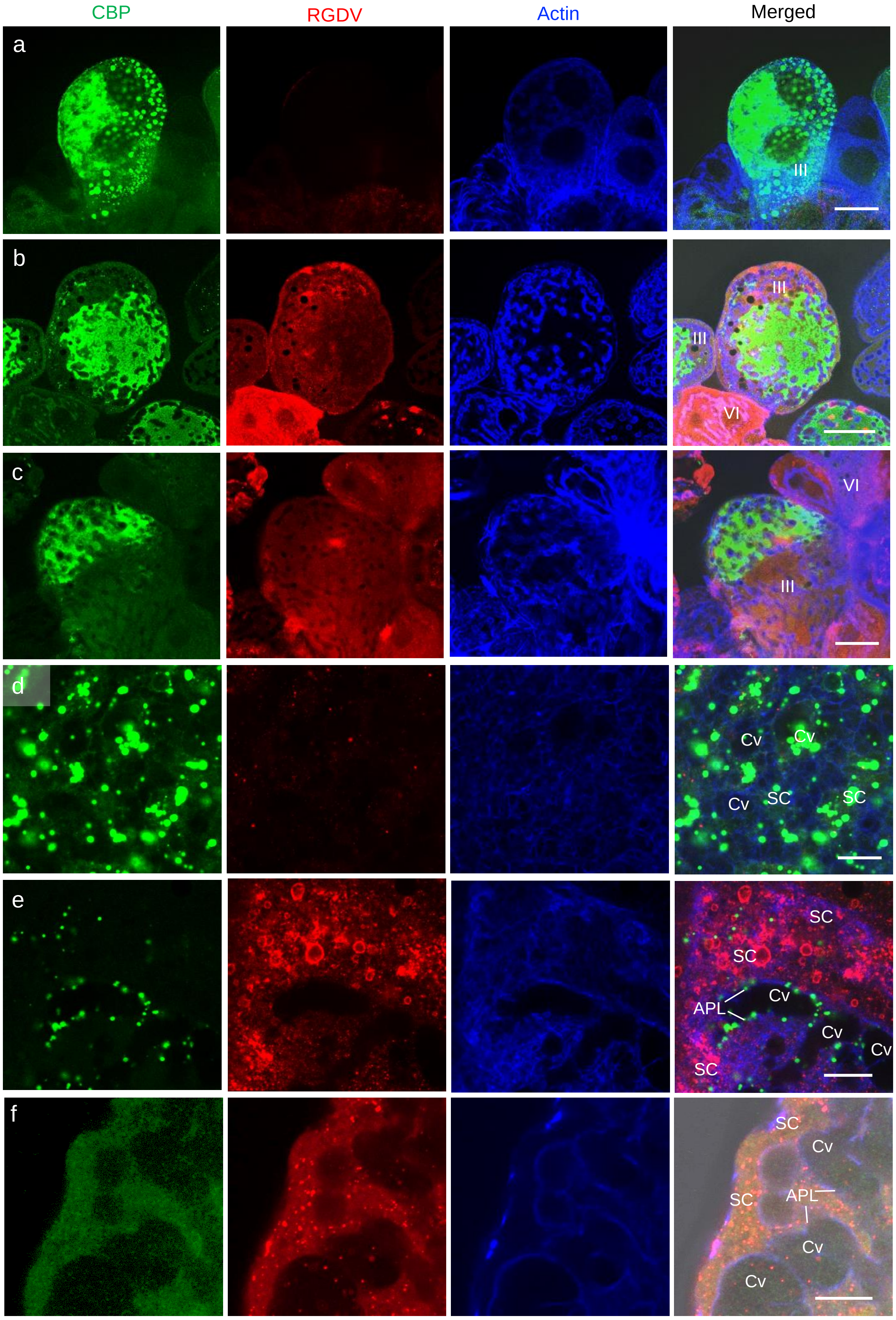

Supplementary Figure 3. Immunofluorescence assay showing that the distribution areas of RdCBP were gradually reduced along with viral infection process in the secretory cells of salivary glands. (a-c) Immunofluorescence assay showing that the absence of colocalization between RdCBP and RGDV virions in salivary gland III-secretory cells after 3 (a). 7 (b) and 9 (c) days post-microinjection of purified viruses, respectively. Split channel images for Figure 2a-c. (d-f) Immunofluorescence assay showing the absence of co-localization between RdCBP and RGDV in salivary gland III-secretory cells after 3 (c). 7 (d) and 9 (e) days post-microinjection of purified viruses, respectively. Salivary glands were immunostained with RGDV P8-rhodamine (red), RdCBP-FITC (green), and the actin dye phalloidin-633 (blue), respectively. Split channel images for Figure 2g and h. Bars: a–c, 50 μm; d–f, 10 μm.

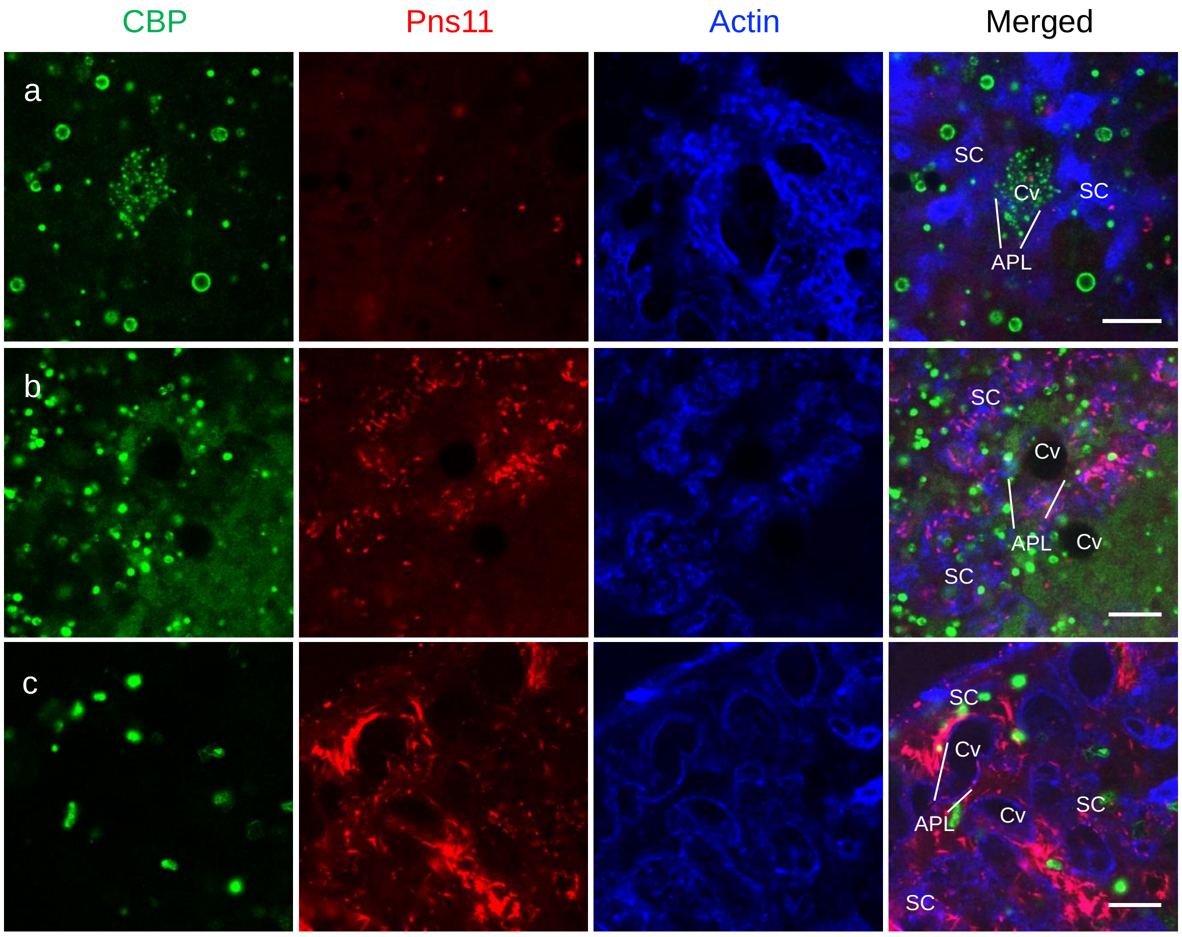

Supplementary Figure 4. Immunofluorescence assay showing the release of RGDV Pns11 filaments and RdCBP into salivary cavities during viral infection of vector salivary glands. Virus-infected salivary glands were dissected from insects after 3 (a), 7 (b), and 9 (c) days post-microinjection of purified viruses. Salivary glands were immunostained with Pns11-rhodamine (red), RdCBP-FITC (green) and phalloidin-Alexa Fluor 647 (blue). Split channel images for Figure 2n-p. Bars, 10 μm.

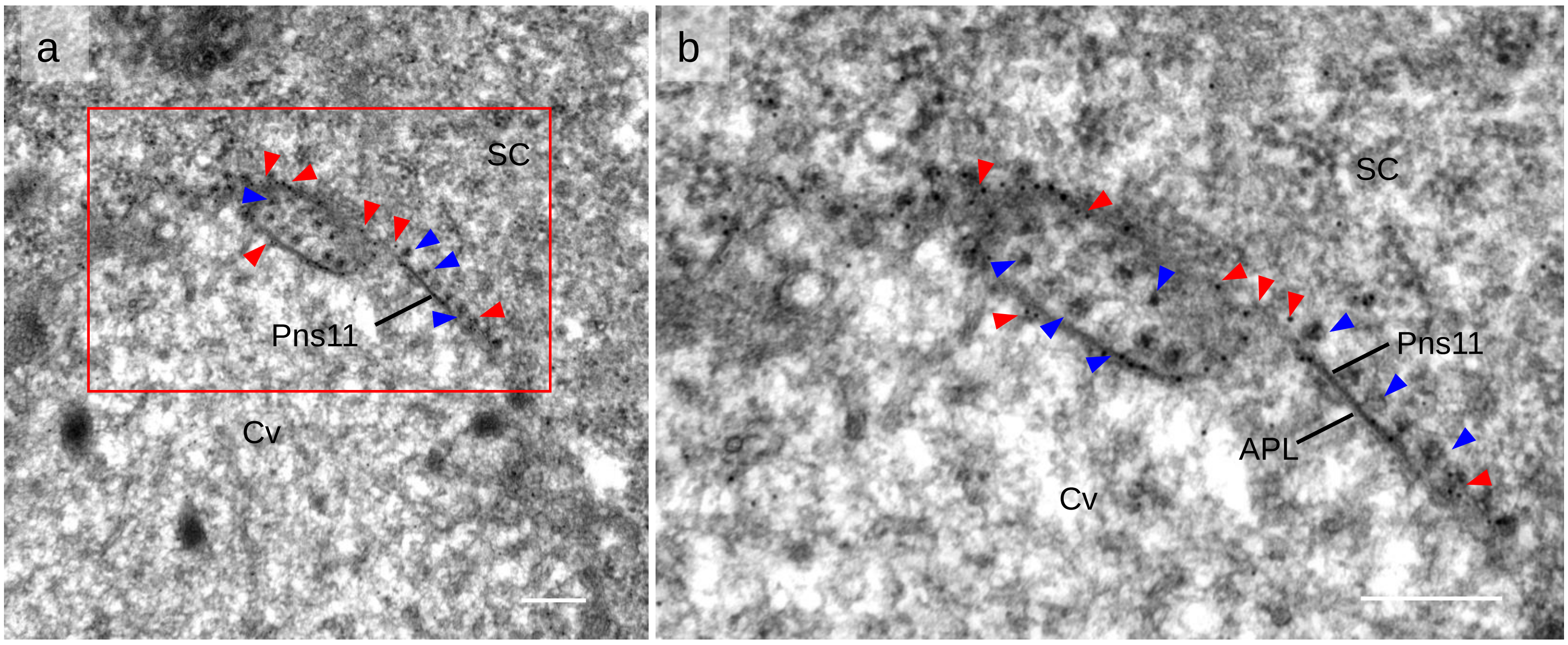

Supplementary Figure 5. Immunoelectron micrographs showing the association of Pns11 of RGDV with actin-based cavity plasmalemma in virus-infected PSG regions. PSGs were immunolabeled with Pns11-specific IgG as primary antibodies, followed by treatment with goat anti-rabbit IgG conjugated with 15-nm-diameter gold particles as a secondary antibody. Red arrows mark gold particles. Blue arrows mark viral particles. (b) is the enlarged field of the boxed area in (a). APL, apical plasmalemma; Cv, cavity; SC, salivary cytoplasm. Bars, 250 nm

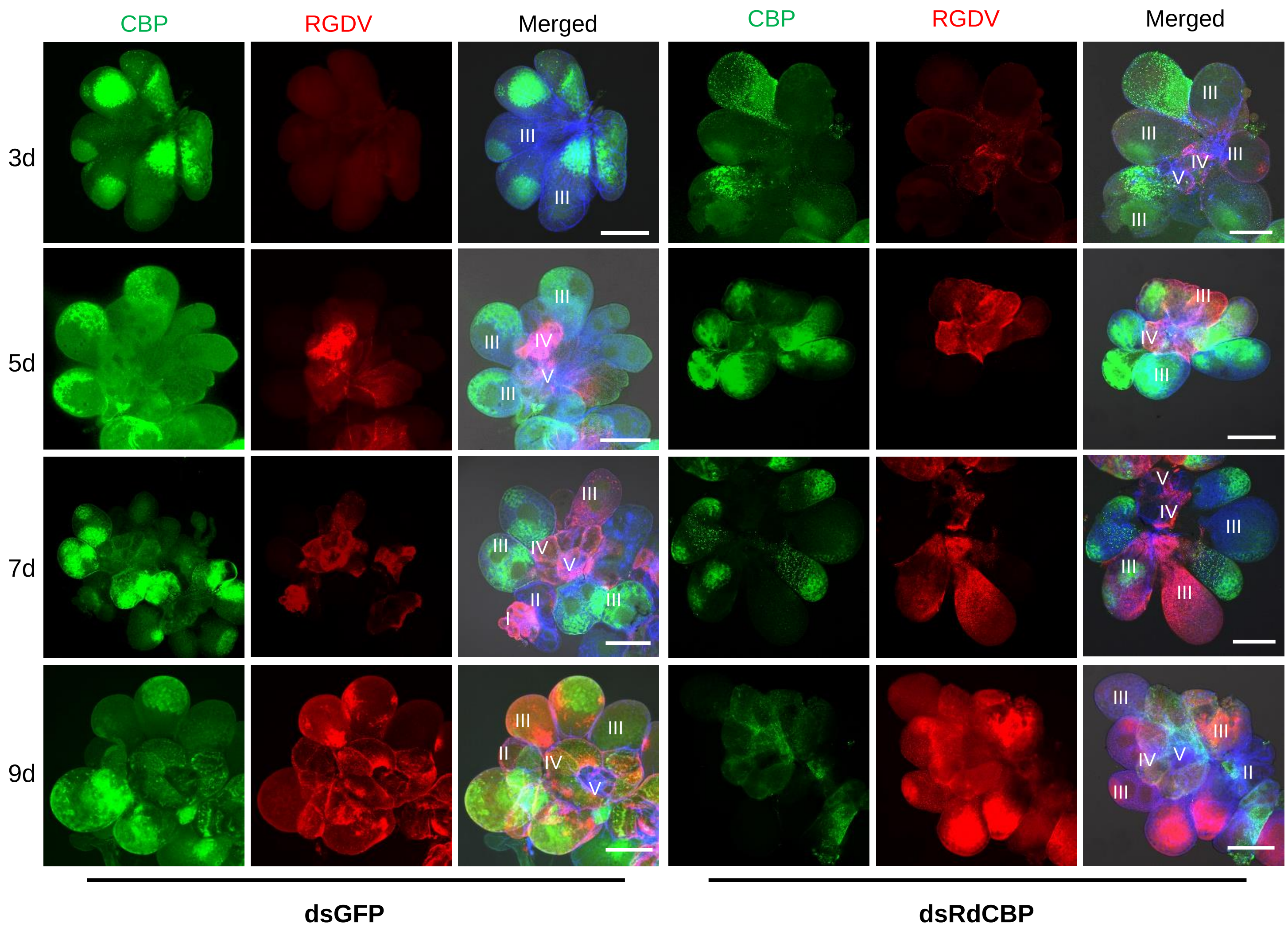

Supplementary Figure 6. Immunofluorescence assay showing the distribution of RdCBP and RGDV in the salivary glands of *R. dorsalis* at different days post-microinjection of a mixture of purified viruses and dsRNAs. Salivary glands were immunostained with virus-rhodamine (red), RdCBP-FITC (green) and phalloidin-Alexa Fluor 647 (blue), and then processed for immunofluorescence microscopy. Split channel images for Figure 4a. Bars, 100 μm.

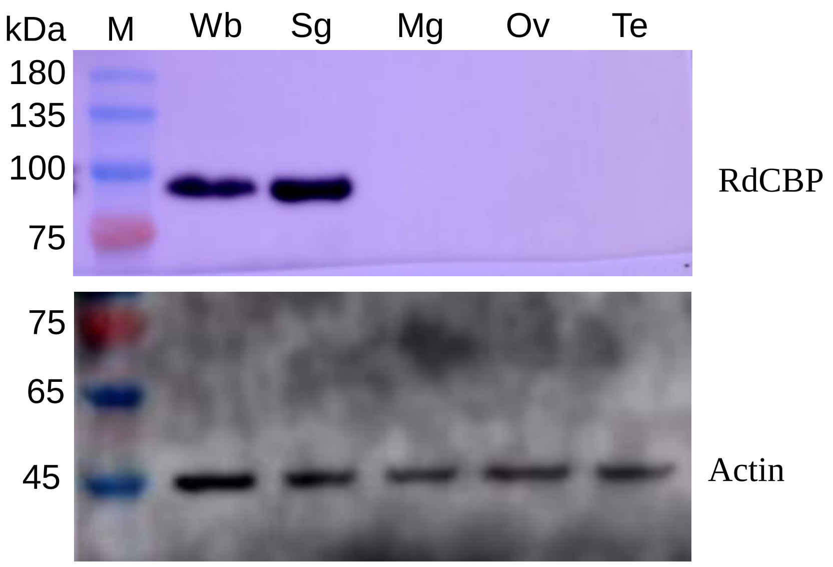

Fig. 1c

Supplementary Figure 7. Original images for immunoblots shown in Figures 1

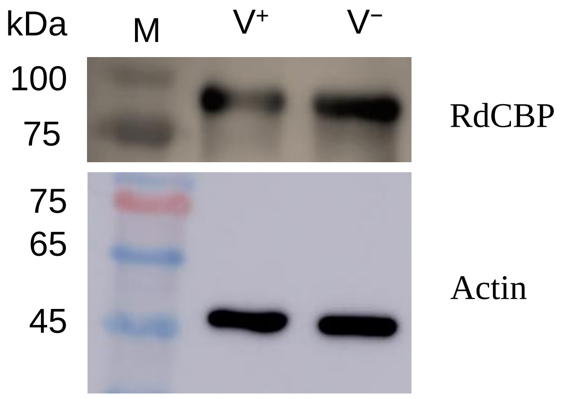

Fig. 2b

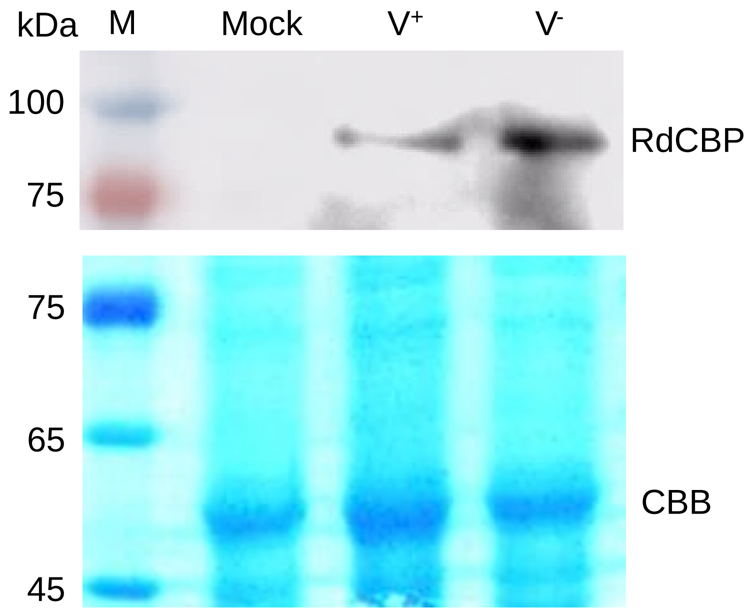

Fig. 2j

Supplementary Figure 8. Original images for immunoblots shown in Figures 2

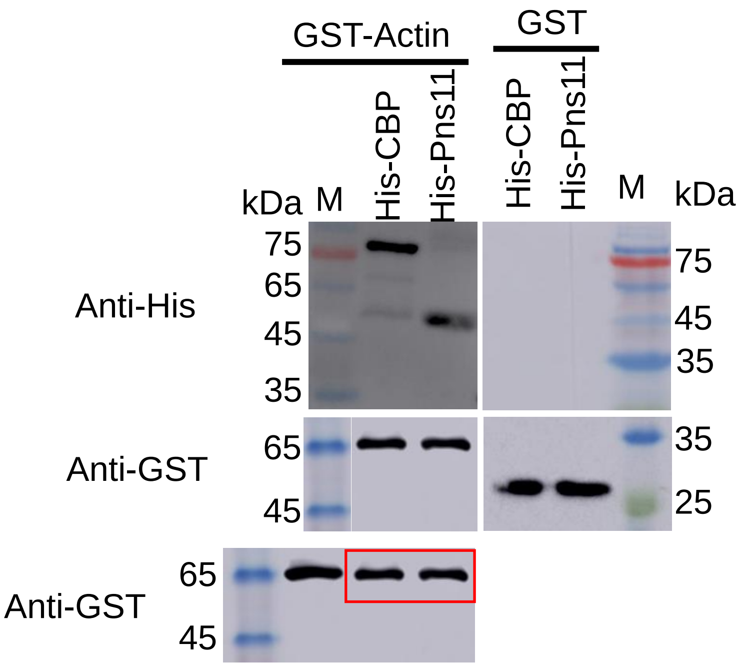

Fig. 3b

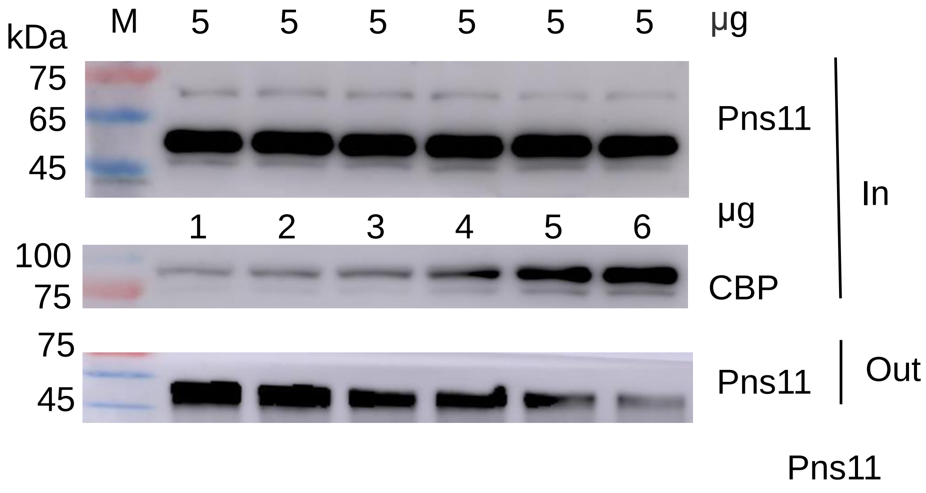

Fig. 3c

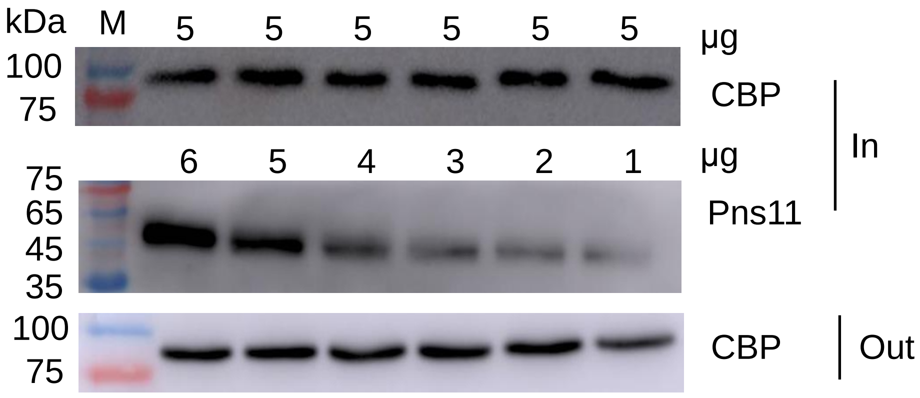

Fig. 3d

Supplementary Figure 9. Original images for immunoblots shown in Figures 3

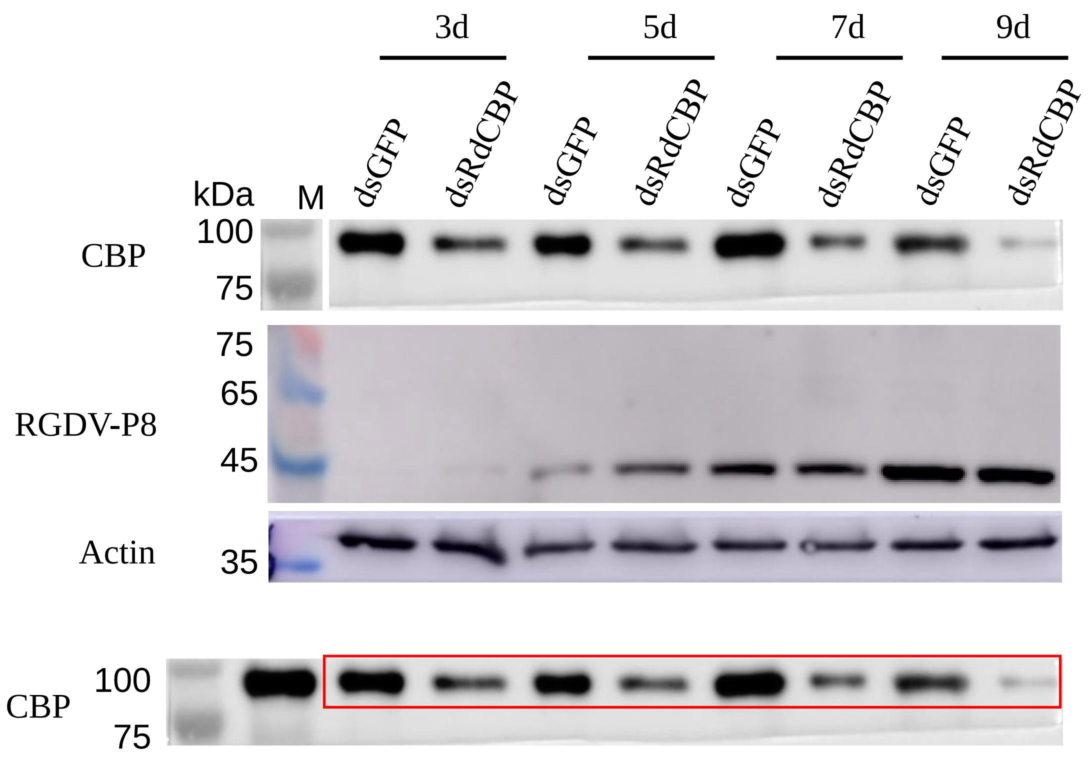

Fig. 4d

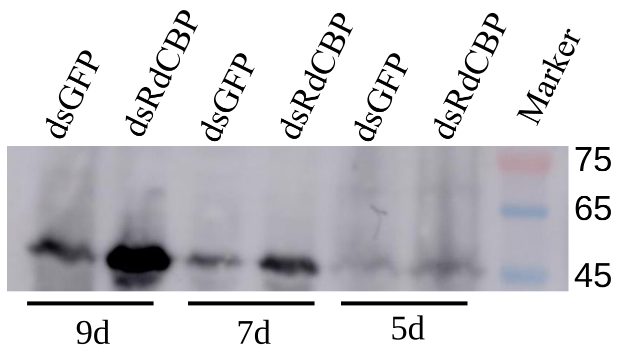

Fig. 4e

Supplementary Figure 10. Original images for immunoblots shown in Figures 4

Supplementary Table 1. Primers used in this study

Oligonucleotide	Assay	Sequence (5’-3’)
EF1_F	qPCR	CAGTGAGAGCCGTTTTGAG
EF1_R	qPCR	AGGGCATCTTGTCAGAGGGC
RGDV_P8_F	qPCR	TGACCTTCATCGTCTCTGAGTCCGA
RGDV_P8_R	qPCR	CGTTACCATTAACCGCGTTCACCTG
RdCBP_F	qPCR	CAAGGCGGCAACTTGGTATC
RdCBP_R	qPCR	CATATGAGACGCGGGGTTGG
T7_RdCBP_F	RNAi	GGATCCTAATACGACTCACTATAGGGACGAC AATAAGGACGACAACA
T7_RdCBP_R	RNAi	GGATCCTAATACGACTCACTATAGGTCAATGG CACGGAACAAGAC
T7_GFP_F	RNAi	GGATCCTAATACGACTCACTATAGGGAAGTTC ACCTTGATGCCGTT
T7_GFP_R	RNAi	GGATCCTAATACGACTCACTATAGGAAGTTCA GCGTGTCCGGCGA
Pns11_F	Y2H	ATTAACAAGGCCATTACGGCCATGGATGCGGA CACTGAGCG
Pns11_R	Y2H	TTGACTAAGGCCGAGGCGGCCGCTAGCTTTC AAGCTTCAATGATTCTAA
RdCBP_F	Y2H	ATTAACAAGGCCATTACGGCCATGGTAACTGA CATCTTGGAAACGAA
RdCBP_R	Y2H	TTGACTAAGGCCGAGGCGGCCGTACTGTGAT TTGGGAGTCAAAGC
Actin_F	Y2H	ATTAACAAGGCCATTACGGCCATGTGTGACGA CGAAGTTGCTG
Actin-R	Y2H	TTGACTAAGGCCGAGGCGGCCGTTAGAAGCA CTTCCTGTGCACG
pDEST_Pns11_F	Pull down	GGGACAAGTTTGTACAAAAAAGCAGGCTTC ATGGATGCGGACACTGAGCG
pDEST_Pns11_R	Pull down	GGGGACCACTTTGTACAAGAAAGCTGGGTCC TAGCTTTCAAGCTTCAATGATTCTAA
pDEST_RdCBP_F	Pull down	GGGACAAGTTTGTACAAAAAAGCAGGCTTCAT GGTAACTGACATCTTGGAAACGAAGG
pDEST_RdCBP_R	Pull down	GGGGACCACTTTGTACAAGAAAGCTGGGTCC TACTGTGATTTGGGAGTCAAAGC
pGEX_Actin_F	Pull down	TGATCGAAGGTCGTGGGATCCCCATGTGTGA CGACGAAGTTGCTG
pGEX_Actin_R	Pull down	TGATCGAAGGTCGTGGGATCCCCTTAGAAGC ACTTCCTGTGCACG
