# Supplementary material for: A leafhopper saliva protein mediates horizontal transmission of viral pathogens from insect vectors into rice phloem
Source: Commun Biol. 2022 Mar 4;5:204. doi: 10.1038/s42003-022-03160-y (PMC8897447; doi:10.1038/s42003-022-03160-y)
Supplement: Supplementary file 5 — Reporting Summary [file 42003_2022_3160_MOESM5_ESM.pdf]

nature portfolio

Corresponding author(s): Taiyun Wei

Last updated by author(s): Nov 20, 2021

Reporting Summary

Nature Portfolio wishes to improve the reproducibility of the work that we publish. This form provides structure for consistency and transparency in reporting. For further information on Nature Portfolio policies, see our Editorial Policies and the Editorial Policy Checklist.

Statistics

For all statistical analyses, confirm that the following items are present in the figure legend, table legend, main text, or Methods section.

n/a	Confirmed	
☐	☒	The exact sample size (n) for each experimental group/condition, given as a discrete number and unit of measurement
☐	☒	A statement on whether measurements were taken from distinct samples or whether the same sample was measured repeatedly
☐	☒	The statistical test(s) used AND whether they are one- or two-sided<br>*Only common tests should be described solely by name; describe more complex techniques in the Methods section.*
☐	☒	A description of all covariates tested
☐	☒	A description of any assumptions or corrections, such as tests of normality and adjustment for multiple comparisons
☐	☒	A full description of the statistical parameters including central tendency (e.g. means) or other basic estimates (e.g. regression coefficient) AND variation (e.g. standard deviation) or associated estimates of uncertainty (e.g. confidence intervals)
☒	☐	For null hypothesis testing, the test statistic (e.g. F, t, r) with confidence intervals, effect sizes, degrees of freedom and P value noted<br>*Give P values as exact values whenever suitable.*
☒	☐	For Bayesian analysis, information on the choice of priors and Markov chain Monte Carlo settings
☒	☐	For hierarchical and complex designs, identification of the appropriate level for tests and full reporting of outcomes
☒	☐	Estimates of effect sizes (e.g. Cohen's d, Pearson's r), indicating how they were calculated

Our web collection on statistics for biologists contains articles on many of the points above.

Software and code

Policy information about availability of computer code

Data collection	*Provide a description of all commercial, open source and custom code used to collect the data in this study, specifying the version used OR state that no software was used.*
Data analysis	Statistical analyses were conducted using SPSS(version 17.0, USA)?Fluorescence intensity was calculated by ImageJ software

For manuscripts utilizing custom algorithms or software that are central to the research but not yet described in published literature, software must be made available to editors and reviewers. We strongly encourage code deposition in a community repository (e.g. GitHub). See the Nature Portfolio guidelines for submitting code & software for further information.

Data

Policy information about availability of data

All manuscripts must include a data availability statement. This statement should provide the following information, where applicable:

- Accession codes, unique identifiers, or web links for publicly available datasets
- A description of any restrictions on data availability
- For clinical datasets or third party data, please ensure that the statement adheres to our policy

Provide your data availability statement here.

March 2021

Field-specific reporting

Please select the one below that is the best fit for your research. If you are not sure, read the appropriate sections before making your selection.

☒ Life sciences ☐ Behavioural & social sciences ☐ Ecological, evolutionary & environmental sciences

For a reference copy of the document with all sections, see nature.com/documents/nr-reporting-summary-flat.pdf

Life sciences study design

All studies must disclose on these points even when the disclosure is negative.

Sample size	Multiple biological replicates were used in all experiments. Exact sample size is provided in each figure legend.
Data exclusions	No data were excluded from analysis
Replication	All experiments were repeated three times for confirmation
Randomization	Insects and plants used in the study were randomly allocated to experimental groups
Blinding	Investigators were not blinded to group assignments

Reporting for specific materials, systems and methods

We require information from authors about some types of materials, experimental systems and methods used in many studies. Here, indicate whether each material, system or method listed is relevant to your study. If you are not sure if a list item applies to your research, read the appropriate section before selecting a response.

Materials & experimental systems

n/a	Involved in the study	
☐	☒	Antibodies
☒	☐	Eukaryotic cell lines
☒	☐	Palaeontology and archaeology
☐	☒	Animals and other organisms
☒	☐	Human research participants
☒	☐	Clinical data
☒	☐	Dual use research of concern

Methods

n/a	Involved in the study	
☒	☐	ChIP-seq
☒	☐	Flow cytometry
☒	☐	MRI-based neuroimaging

Antibodies

Antibodies used	His antibody (Sigma, cat.SAB4301134); GST antibody (Sigma, cat. SAB4200692); Actin antibody(Sigma, cat. SAB4502543); Anti-Rabbit IgG –Peroxidase (Sigma, cat.A0545); Anti-Mouse IgG–Peroxidase(Sigma, cat.A9309);Anti-Mouse IgG–Gold (Sigma, cat.G7652); Anti-Rabbit IgG–Gold (Sigma, cat.G7402);Antibodies against RdCBP was produced by Genscript USA Innovation Company (Nanjing); Antibodies against RGDV intact virions and Pns 11 were prepared in our laboratory.
Validation	The validation statements of commercial antibodies are available on the manufacturer's website. The validation of antibodies prepared in our laboratory is provide in this manuscript or as part of previously published studies.

Animals and other organisms

Policy information about studies involving animals; ARRIVE guidelines recommended for reporting animal research

Laboratory animals	The leafhopper Recilia dorsalis was used in this stud
Wild animals	The study did not involve wild animals.
Field-collected samples	The field-collected leafhopper Recilia dorsaliswas used in this study and the relevant parameters were provided in the manuscript
Ethics oversight	No ethical approval was required, the insects used in the study are agricultural pests.

Note that full information on the approval of the study protocol must also be provided in the manuscript.
